# Rapid diagnosis of Lemierre’s syndrome by metagenomic next-generation sequencing: a case report

**DOI:** 10.3389/fmed.2025.1730031

**Published:** 2026-01-02

**Authors:** Yanhui Wang, Xinqi Zhang

**Affiliations:** Department of Emergency, the 960th Hospital of the People’s Liberation Army, Shandong Second Medical University (The 960th Hospital of the PLA Joint Logistics Support Force), Jinan, Shandong, China

**Keywords:** case report, diagnose, *Fusobacterium necrophorum*, Lemierre’s syndrome, metagenomic next-generation sequencing

## Abstract

Lemierre’s syndrome, also known as postopharyngeal septicaemia or necrobacillosis, is a rare, fatal opportunistic infection, often caused by *Fusobacterium necrophorum* invading the throat. Bacterial culture is a conventional method to establish a diagnosis, but is time-consuming and insensitive in some cases. Metagenomic next-generation sequencing (mNGS), as an emerging technique, has become an important supplementary detection method for infectious diseases. It greatly favors the rapid, precise diagnosis and treatment of Lemierre’s syndrome through accurately obtaining etiological information. We reported a case of Lemierre’s syndrome that was rapidly and accurately diagnosed by mNGS.

## Introduction

Lemierre’s syndrome, also known as postopharyngeal septicaemia or necrobacillosis, is a rare but life-threatening condition that mostly affects healthy male adults. It was initially reported by Professor Lemierre in a series of 20 cases (1). Although rare with a global incidence of 1 case per 1,000,000 people, the mortality of Lemierre’s syndrome can be up to 5–18% (2). It is manifested as infectious thrombophlebitis of the internal jugular vein secondary to acute oropharyngeal infection. In the lungs, the most involved site of metastatic infection, Lemierre’s syndrome presents with pulmonary abscesses, pleural effusion, empyema, pulmonary bulla and pneumothorax (3). *Fusobacterium necrophorum* is mainly responsible for Lemierre’s syndrome (4–6). An etiological diagnosis can be made through bacterial culture, which is time-consuming and less sensitive. At present, metagenomic next-generation sequencing (mNGS) has emerged to effectively and accurately identifies the etiology of infectious diseases. In the present study, we reported a rare case mNGS-diagnosed with Lemierre’s syndrome caused by *Fusobacterium necrophorum* infection.

## Case presentation

A 23-year-old male patient presented in the emergency department for 6 days of pharyngeal pain, cough, and phlegm production. He complained of a sudden sore throat 6 days earlier, especially in swallowing, after getting cold, accompanied by paroxysmal coughing up with white, foamy mucus, and difficulty in expelling phlegm. Symptoms could not be relieved by oral medications of anti-inflammatory and cough suppressant tablets, Pudilan Xiaoyan oral liquid (PDL), and compound paracetamol and amylamine tablets. He developed fever 2 days earlier, with a maximum self-tested temperature of 39.3 °C. Testing was negative for COVID-19, influenza A virus, and influenza B virus in a local hospital. Laboratory testing showed elevated inflammatory cell levels. Chest computed tomography (CT) visualized infection and consolidation in the right lower lung. The patient was diagnosed with pulmonary infection, and managed by an anti-infection therapy regimen of intravenous (iv) administration of cefazolin sodium 1.0 g for a total of 5 days.

The patient asked for further treatment in our hospital due to unrelieved symptoms. His physical condition was good. Physical examinations on admission showed a temperature of 36.7 °C, a blood pressure of 78/48 mmHg (1 mmHg = 0.133 kPa), a pulse rate of 76 beats per minute (bpm), a respiratory rate of 15 bpm, and a blood oxygen saturation of 96% (without oxygen inhalation). The skin of the extremities was pale and cold. Bilateral tonsils were congested, red, swollen, and enlarged (Grade 2 hypertrophy), presenting white purulent secretions and centered uvula. A rough breathing sound in the right lung, and dry and slight moist rales were auscultated in the lower right lung.

**Figure 1:**
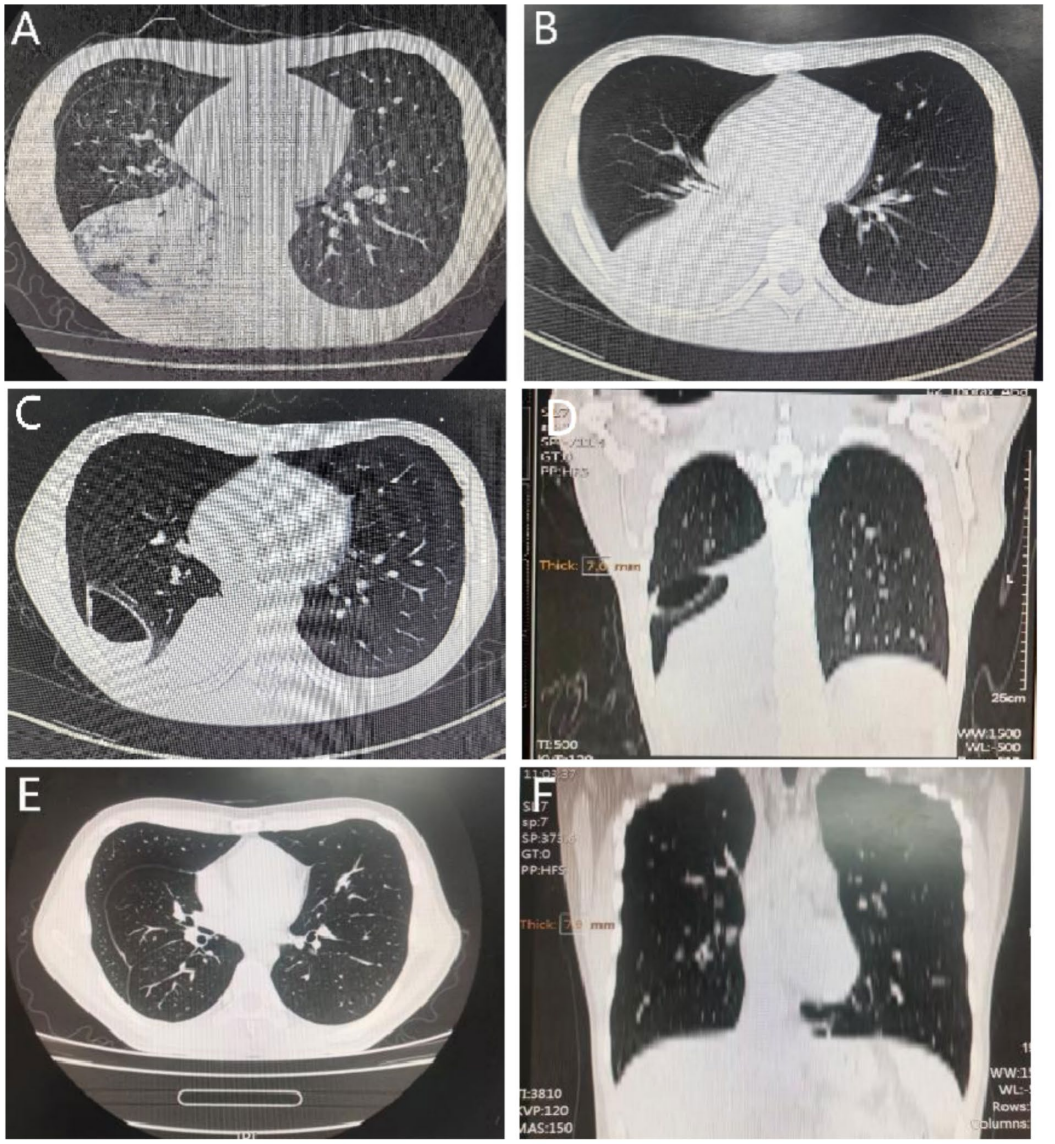
Chest CT images of the patient at different stages. Chest CT scans on May 21, 2022 **(A)**, and May 25, 2022 **(B)**. Chest CT on May 31, 2022 in transverse **(C)** and coronal views **(D)**. Reexaminations of chest CT with the axial **(E)** and coronal views **(F)**.

**Figure 2:**
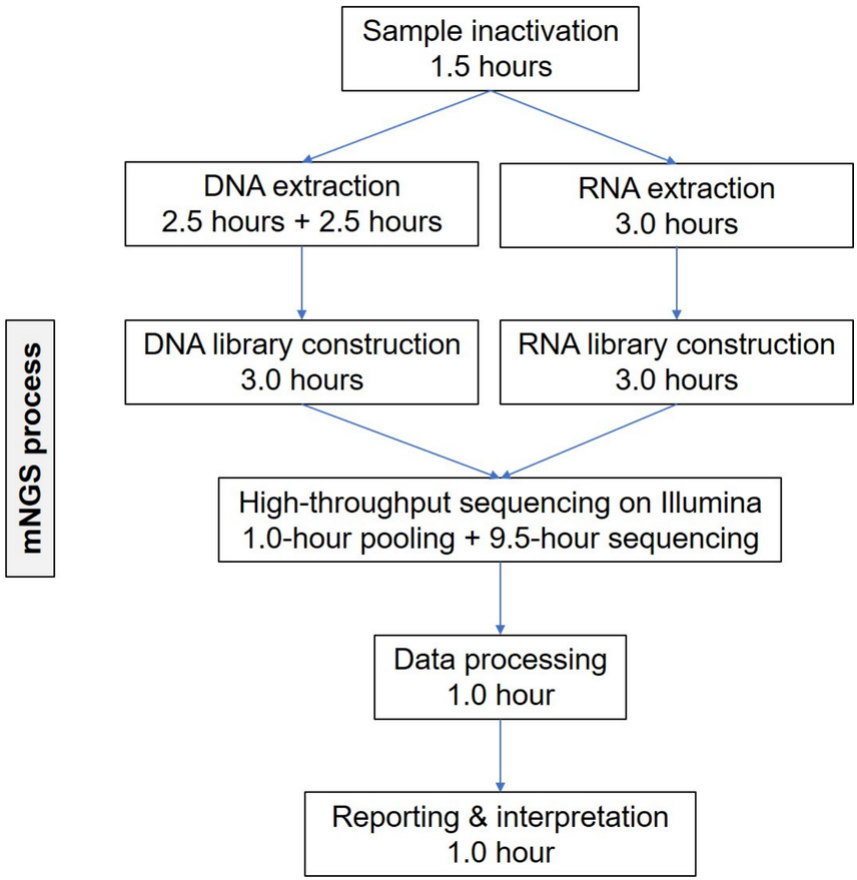
Workflow of mNGS.

Abnormal laboratory testing findings included: WBC, 14.46 × 10^9^/L ↑; neutrophil percentage (NEU%), 91.5% ↑; hemoglobin (Hb), 127 g/L ↓; red cell count (RBC), 4.16 × 10^12^/L ↓; CRP, 276.94 mg/L ↑; procalcitonin (PCT), 2.33 ng/mL ↑; D-dimer, 0.63 mg/L ↑. Pathological examination was not conducted in the local hospital. Considering the critical condition, the patient was examined in the emergency department immediately with chlamydia antibody, mycoplasma, tuberculosis antibody, and β-D-glucan (G test) and galactomannan (GM test) assays, all of which were negative. Bacteria were not detected in sputum culture. Chest CT on May 21, 2022 showed infections in the right lung, and small nodules in both lungs (Figure 1A). The patient was preliminarily diagnosed with pneumonia and sepsis, and managed by an anti-infection treatment (piperacillin, tazobactam and ulinastatin) and symptomatic measures to raise the blood pressure. Chest CT on May 25, 2022 showed aggravated infections in the right lung, small nodules in both lungs, a small pericardial effusion, and bilateral pleural effusion (Figure 1B). On the second day of admission, mNGS of cell-free DNA samples was conducted in Simcere Medical Diagnosis Co., Ltd., Jiangsu, China, briefly through a multi-step process of sample inactivation, DNA/RNA extraction, library construction, high-throughput sequencing on Illumina, bioinformatics analysis and report generation (Figure 2). Finally, *Fusobacterium necrophorum*, as the pathogenic bacteria, was finally determined by mNGS on the blood samples (Table 1).

**Table 1:** Blood sample report of mNGS.

	Genus			Species	
	Latin name	Count of reads	Abundance	Latin name	Count of reads
Gram-negative bacteria	Fusobacterium	26	1.53%	*Fusobacterium necrophorum*	24
Gram-positive bacteria	Not found				
Fungus	Not found				
Virus	Not found			HHV-4	11
	Not found			TTV	5
Parasite	Not found				
MCT	Not found				

HHV-4, human gammaherpesvirus 4; TTV, Torque teno virus; MCT, *Mycobacterium tuberculosis* complex.

Clinical symptoms were significantly relieved by intravenous administrations of moxifloxacin (0.4 g, once a day, for 4 days) and piperacillin sodium/sulbactam sodium (4.5 g, every 8 h, for 8 days). The dosage of metaraminol (0.3 mg/min) to raise the blood pressure was gradually reduced. Chest CT on May 31, 2022 showed a smaller range of consolidation in the right lower lung than before, a small amount of pericardial effusion, bilateral pleural effusion, and air and blood in the right interlobular pleural space, suggesting the presence of inflammation combined with atelectasis and pneumothorax (Figures 1C,D). The mNGS report of *Fusobacterium necrophorum* provided a clue for the diagnosis of Lemierre’s syndrome. Bedsides, color Doppler ultrasonography further suggested thrombophlebitis of the internal jugular vein (Figure 2). The patient asked for discharge due to work obligations. One month later, reexamination of the chest CT with the axial (Figure 1E) and coronal views (Figure 1F) in the local hospital did not show abnormalities. A summary of vital signs, laboratory testing, and use of antibiotics and vasopressors during the treatment period is shown in Table 2.

**Table 2:** A summary of vital signs, laboratory testing, and use of antibiotics and vasopressors during the treatment period.

Admission day						
Indicators	1 day	2 days	4 days	6 days	8 days	12 days
WBC (×10^9^/L)	14.46	18.64	17.96	17.9	11.63	8.97
PCT (μg/L)	2.33	0.66	0.18	0.11	0.08	0.06
CRP (mg/L)	276.9	123.0	41.3	34.1	6.36	1.6
D-D (mg/L)	0.63	0.89	1.85	6.85	2.28	0.64
BP (mmHg, 1 mmHg = 0.133 kPa)	76/57	85/62	93/68	101/71	115/82	128/93
Metaraminol dose	0.3 mg/min	0.2 mg/min	0.2 mg/min	0.1 mg/min	Withdrawal	Withdrawal
Antibiotics	Intravenous administration of piperacillin sodium/sulbactam sodium (4.5 g, every 8 h, for 8 days)	Intravenous administration of piperacillin sodium/sulbactam sodium (4.5 g, every 8 h, for 8 days)	Intravenous administration of piperacillin sodium/sulbactam sodium (4.5 g, every 8 h, for 8 days)	Intravenous administration of piperacillin sodium/sulbactam sodium (4.5 g, every 8 h, for 8 days)	Intravenous administrations of piperacillin sodium/sulbactam sodium (4.5 g, every 8 h, for 8 days) plus moxifloxacin (0.4 g, once a day, for 4 days)	Intravenous administrations of piperacillin sodium/sulbactam sodium (4.5 g, every 8 h, for 8 days) plus moxifloxacin (0.4 g, once a day, for 4 days)

WBC, white blood cell; PCT, procalcitonin; CRP, C-reactive protein; D-D, D-dimer; BP, blood pressure.

## Discussion

**Figure 3:**
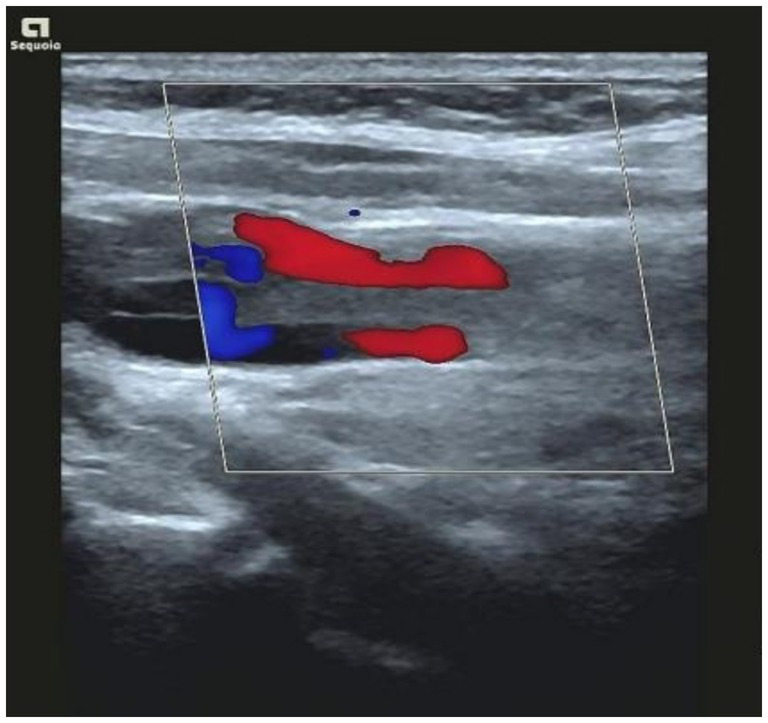
Bedside color Doppler ultrasonography suggested thrombophlebitis of the internal jugular vein.

Previously, a diagnosis of Lemierre’s syndrome by mNGS has not been reported. Existing reports only described the application of mNGS to the detection of *Fusobacterium necrophorum* (7) (see Figure 3).

Lemierre’s syndrome, as a rare condition, usually invades young adults (3, 8), with an incidence lacking estimates. It is diagnosed based on the following three criteria: (1) an infection mainly in the head and neck region, usually accompanying a history of sore throat or pharyngeal infection; (2) thrombosis or thrombophlebitis in the internal jugular vein or other veins in the head and neck, or bacterial metastases; (3) presence of *Fusobacterium necrophorum* in blood cultures or other normally sterile sites (8). At present, whether an oropharyngeal infection, rather than any infection in the head and neck region, is a necessary condition for diagnosing Lemierre’s syndrome remains controversial. Atypical or variant Lemierre’s syndrome refers to a condition stemming from primary infections outside of the head and neck region. In addition, existing evidence also suggests that internal jugular venous thrombosis is not a mandatory diagnostic criterion for Lemierre’s syndrome. The presence of internal jugular vein thrombosis can be missed or resolved during the course of disease, even if it exists (9).

We reported a young man with sore throat as the initial manifestation. Bacterial infection and septic shock were supported by bilateral tonsil enlargement, elevated WBC, NEU%, CRP and PCT. Additionally, an elevated D-dimer level indicated the development of thrombosis. Right lung infection was confirmed on chest CT scans. Importantly, mGNS of blood sample confirmed the presence of *Fusobacterium necrophorum*. The patient was finally diagnosed with Lemierre’s syndrome.

Lemierre’s syndrome used to bring with a high mortality before the advent of antibiotics. It can be frequently misdiagnosed, leading to severe complications or even deaths (10). Antibiotics are the top priority in the treatment of Lemierre’s syndrome. Beta-lactamase inhibitors, metronidazole and clindamycin are mainstream beneficial antibiotics (10, 11). While anticoagulation is controversial in treating Lemierre’s syndrome, therapeutic doses might provide clinical benefit to individuals without contraindications (12). An early diagnosis is critical for the prognosis of Lemierre’s syndrome (13). In this case report, mNGS is a powerful tool to rapidly identify *Fusobacterium necrophorum*, and accurately confirm the diagnosis of Lemierre’s syndrome. Our clinical experience may support the use of mNGS in the precise diagnosis and early treatment of Lemierre’s syndrome.

For individuals with typical manifestations of Lemierre’s syndrome (e.g., acute onset of high fever, neck pain, sepsis, and metastatic infections), unstable vital signs and poor responses to common antibiotics, the mNGS of blood samples combined with sputum culture is recommended to make an accurate diagnosis.

## Data Availability

The original contributions presented in the study are included in the article/supplementary material, further inquiries can be directed to the corresponding author.
